# Electrically conductive, hydrophobic, UV protective and lightweight cotton spunlace nonwoven fabric coated with reduced graphene oxide

**DOI:** 10.55730/1300-0527.3408

**Published:** 2022-02-23

**Authors:** Bekir Cenkkut GÜLTEKİN

**Affiliations:** Department of Textile Engineering, Faculty of Technology, Marmara University, İstanbul, Turkey

**Keywords:** Cotton, nonwoven fabric, graphene oxide, coating, reduction

## Abstract

In this study, cotton spunlace nonwoven fabrics with four different basis weights were coated with graphene oxide (GO) by conventional dip coating method which is one of the most simple and effortless process in textile industry. The graphene oxide coated cotton nonwoven fabrics were immersed in an aqueous sodium dithionite solution in order to obtain reduced graphene oxide (RGO) coated cotton spunlace nonwoven fabrics. The obtained nonwoven fabrics became electrically conductive with very low surface electrical resistivity of 5.98 × 10^2^ W/sq for the 70 g/m^2^ basis weight nonwoven fabric. The color measurements and reflectance spectrophotometry were performed in order to identify the coating and reduction process of GO on cotton nonwoven fabrics. The hydrophobic characteristic of the GO and RGO coated cotton nonwoven fabrics were determined by the means of water contact angle. The ultraviolet (UV) blocking ability of the cotton nonwoven fabric both coated with GO and RGO were analyzed by the UV transmittance analyzer. The water contact angle results revealed that the hydrophilic cotton nonwoven fabric became hydrophobic due to the reduction of GO. It was also found that the ultraviolet protection factor (UPF) depends on the basis weight of the cotton nonwoven fabric and the reduction of GO.

## 1. Introduction

Functional textile materials have attracted great attention for their electrical conductivity [1, 2], antibacterial [3, 4], fire retardant [5, 6], superhydrophobic [7, 8], ultraviolet (UV) blocking [9, 10], energy storage [11], sensing properties [12, 13]. These textiles have potential applications in biomedical monitors, high performance textiles, sportswear, wearable displays, military garments [14, 15]. Functional textiles can be prepared by approaches such as spinning, knitting, weaving, coating, and printing. Cotton is the predominant natural fiber in the textile industry because of its natural softness, high hygroscopicity, superior wear comfort and skin-friendliness [16]. Nonwoven fabrics are one of the fastest growing part of textiles of the global textile industry [17]. The nonwoven fabrics have large number of applications such as sound absorption [18], reinforcing fibers [19], healthcare [20, 21], filter media [22], electromagnetic shielding [23, 24] and membrane [25], etc. Therefore, nonwoven fabric can be an ideal supporting material for functional textiles, in particular for electrically conductive textiles. Spunlace nonwoven fabrics are manufactured through entangling loose fiber webs by using jets of water. High velocity jets of water are passed through a closely spaced nozzle to mechanically interlock fibers through displacement, twisting and rearrangement or reorientation to create fabrics [26]. This process provides unique nonwoven fabrics without damaging fibers and without the need for a binder [27]. In the manufacturing process of spunlace nonwoven fabrics, short staple fibers with an average fiber length of 38–40 mm are used [28]. Cotton nonwoven fabric possess a hierarchical structure with high porosity, large surface area and hydrophilic functional groups [14] and also, have lots of advantages such as low cost, light weight, environmentally friendliness [29].

Graphene, one of the allotropes (carbon nanotube, fullerene, diamond) of elemental carbon, is a planar monolayer of carbon atoms arranged into a 2-dimensional (2D) honeycomb lattice [30, 31]. Graphene has attracted great attention because of its excellent properties, such as high thermal and electrical conductivity [32]. Chemical conversion of graphite to graphene oxide (GO) that is a process with low cost and high reaction yield, has emerged to be a viable route to obtain graphene or reduced graphene oxide [30, 33]. Compared to pristine graphite, GO is heavily oxygenated hydroxyl and epoxy groups on the basal plane, in addition to carbonyl and carboxyl groups located at the sheet edges [30, 34]. Hence, GO is highly hydrophilic and readily exfoliated in water, yielding stable dispersion. On the other hand, GO is electrically insulating due to the disruption of the conjugated electronic structure by these functional groups. Chemical reduction of graphene oxide usually takes place to obtain electrically conductive form [35, 36]. GO is more suitable for functional textiles because it can be applied to textiles in various techniques, including dip coating [37], screen printing [38], ink-jet printing [39], dyeing [40]. Regarding these various techniques to produce functional textiles, the most widely used method is the dip coating which is simple, easy to apply and scalable [41]. Recently, several studies were reported on the modification of textiles by coating with graphene oxide for obtaining functional textiles. Mengal et al. [42] produced a stable textile electrode from plain woven lyocell fabric by coating with reduced graphene oxide (RGO). Xu et al. [43] coated the cotton fabric with graphene oxide followed by the reduction process in order to obtain flexible electrode material for electrochemical capacitors. Tian et al. [44] prepared cotton fabric coated with graphene oxide and chitosan via the electrostatic layer-by-layer technique which has ultraviolet (UV) protection property. Tang et al. [9] prepared multifunctional cotton fabric with electrical conductivity and UV protection properties by coating graphene oxide and then synthesizing polyaniline (PANi) by in situ chemical polymerization process. Mizerska et al. [45] obtained the electrical conductivity and hydrophobicity in cotton fabric through a sol-gel method by coating with an organosilicon sol containing graphene oxide followed by reduction of graphene oxide by thermal treatment. Du et al. [46] fabricated graphene based wearable textile strain sensors by coating nonwoven fabric with graphene oxide followed by chemical reduction. Zhou et al. [47] coated cotton fabric with silica (SiO_2_)/reduced graphene oxide (RGO) to obtain functional properties such as asymmetric wettability, air permeability and thermal insulating properties.

There is limited information available in the literature about coating spunlace nonwoven fabrics with graphene oxide and imparting multifunctional properties to the nonwoven fabrics. As mentioned above, cotton spunlace nonwoven fabric is environmental friendly, light weight and low cost material. Also, the nonwoven fabric has large surface area and hydrophilic functional groups which makes it an ideal substrate material to use with graphene oxide. The oxygen containing functional groups both from the GO and cotton nonwoven fabric form hydrogen bonds with each other which enhances the adhesion and allows efficient coating. These functional groups make the dip coating process realizable.

Therefore, the aim of this study is the fabrication of cotton spunlace nonwoven fabric by coating with graphene oxide and then by the reduction of graphene oxide with chemical method to obtain multifunctional nonwoven fabric with electrically conductivity. In textile dyeing process, especially in the coloration of cellulose fibers, vat (including indigo) and sulphur dyes are widely used. Currently, sodium dithionite is the most important reducing agent used in the industrial reduction of vat dyes and sulphur dyes [48–50]. Recently, new fields of application have been developed such as chemical reduction of graphene oxide [40, 51–55]. Chemical reduction method is more suitable for GO coated textile materials than the thermal reduction method due to the process temperature lower than 100 °C. Therefore, chemical reduction of graphene oxide coated cotton spunlace nonwoven fabric was carried out with sodium dithionite as reducing agent. To examine the effect of basis weight of nonwoven fabric, cotton spunlace nonwoven fabrics with four different basis weights were coated with GO and reduction reactions applied. Subsequently, the surface morphology and elemental analysis, chemical structure, tensile strength, color coordinates, surface electrical resistance, water contact angle, UV blocking properties were investigated.

## 2. Experimental

### 2.1. Materials

The 100% cotton spunlace nonwoven fabric with 4 different basis weights (40, 50, 60, and 70 g/m^2^) was kindly supplied by Ihsan Sons Limited, Pakistan. The tensile property of the cotton spunlace nonwoven fabrics was indicated in Table 1 according to the data shared by the supplier. All chemicals were of analytical reagent grade and used without further purification. Graphite flakes were purchased from Sigma Aldrich. Hydrogen peroxide (H_2_O_2_, 35%), sulfuric acid (H_2_SO_4,_ 95%–98%), phosphoric acid (H_3_PO_4_), potassium permanganate (KMnO_4_) and hydrochloric acid (HCl, 37%), sodium dithionite were purchased from Merck. Distilled water was used throughout the experiments.

### 2.2. Synthesis of GO

Graphene oxide was synthesized from flake graphite by the improved Hummer’s method according to Ref. [56]. Briefly, a 9:1 mixture of concentrated H_2_SO_4_/H_3_PO_4_ (360:40 mL) was added to a mixture of graphite flakes (3 g) and KMnO_4_ (18 g). The reaction was then heated to 50 °C and stirred for 12 h. The reaction was then cooled to room temperature and poured onto ice (400 mL) with 35% H_2_O_2_ (6 mL). The resulting suspension was washed by repeated centrifugation (each at 8000 rpm for 30 min), first with 400 mL of 1 M HCl and 200 mL of ethanol (2′), then with distilled water until a pH of 4–5 was achieved. The obtained solid product was dried overnight in an oven at 60 °C.

### 2.3. Coating of cotton nonwoven fabric with GO

The synthesized GO nanosheets were dispersed in distilled water by bath sonication method for about 60 min to obtain 2 mg/mL GO aqueous dispersion. The cotton spunlace nonwoven fabric was dip coated in a bath with liquor to goods ratio (L:G) = 40:1 containing 2 mg/mL GO dispersion for about 30 min at 60 °C. Because of the strong adsorption, the cotton nonwoven was quickly coated by the GO. Then, the coated fabric was kept in an oven at 70 °C for 30 min. The coating process was repeated 5 times in order to increase the GO adsorption on the nonwoven fabric. The obtained fabrics were coded as GONF according to the basis weights. For example, GONF40 corresponds to the 40 g/m^2^ basis weight nonwoven fabric coated with GO.

### 2.4. Reduction of GO coated cotton nonwoven fabric

**Figure 1:**
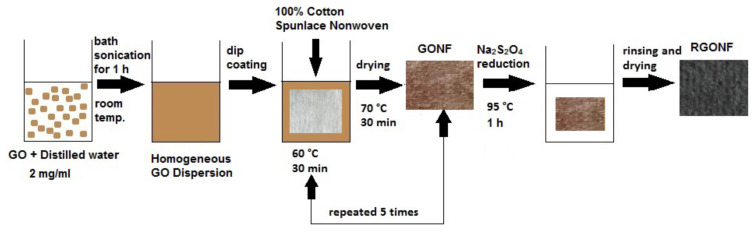
Coating and reduction processes of GO onto cotton nonwoven fabric.

The reduction process of graphene oxide coated cotton nonwoven fabrics was carried out using sodium dithionite (Na_2_S_2_O_4_) as a reducing agent. The GONF samples were immersed into the reduction bath containing 0.1 M aqueous solution of Na_2_S_2_O_4_ for about 60 min. The liquor to goods ratio (L:G) was adjusted as 70:1. The temperature of the reduction process was kept at 95 °C. The resulting fabrics were rinsed with distilled water to remove the remaining reducing agent. The fabrics were dried in an oven at 90 °C. The obtained fabrics after reduction process were coded as RGONF. For example, RGONF40 corresponds to the 40 g/m^2^ basis weight nonwoven fabric coated with reduced GO. The GO coating and reduction process is given schematically in Figure 1.

### 2.5. Characterization

Scanning electron microscopy (SEM, FEI Sirion) was employed to observe the morphology of coated samples. The surfaces of the samples were coated with gold at 1.5 kV for 100 s before analysis. An attenuated total reflection Fourier transform infrared spectrometer (ATR-FTIR, Perkin Elmer Spectrum Two) was used at 450–4000 cm^−1^. The Raman spectra were acquired using a WITec alpha300 RA with a 523 nm laser (WITech, Germany). The mechanical properties were tested with a tensile mechanical tester (Instron 4411) at the machine direction (MD). It is noteworthy to mention that the almost all nonwoven fabrics are anisotropic, having more fiber orientation in the machine direction (MD) than the cross direction (CD) [57]. The specimens were prepared at dimensions of 75 × 50 mm. The crosshead speed was 300 mm/min. Each sample was tested at least five times and the average value was calculated. The tensile strength and elongation at break were determined. Color coordinates were determined by Datacolor SF600+, using SAV aperture and SI mode, and the color differences were calculated in accordance with the CIELab system with D65/10° observer values. In CIE Lab system, the lightness L* represents the darkest black at L* = 0 and the brightest white at L* = 100, a* represents redness (a* is +)/greenness (a* is −) and b* represents yellowness (b* is +)/blueness (b* is −). a* and b* represent true neutral gray values at a* = 0 and b* = 0. The color difference (ΔE) was determined using the following equation [9]:


(1) $$\Delta \text{E}=\sqrt{\Delta {\text{L}}^{*2}+\Delta {\text{a}}^{*2}+\Delta {\text{b}}^{*2}}.$$

The surface electrical resistance of the fabrics was measured using a standard four-point probe.

The setup consists of a sourcemeter (Keithley 2450 Sourcemeter) and a four point probe station (Everbeing Int’l Corp). Each sample was measured at least five times and the average value was calculated. The water contact angle of the cotton nonwoven fabrics was measured by using of a PGX Goniometer (FIBRO Systems, Sweden). In the measurements, deionized water was used as standard liquid that was deposited on the nonwoven fabric surface. The UV protection ability was recorded by a UV transmittance analyzer (UV 1000F, Labsphere, SDL ATLAS). Each sample was measured at least five times and the average value was calculated. The protection activity of a fabric to block ultraviolet light is defined as the ultraviolet protection factor (UPF) value. The test was done under the wavelength range of 290–400 nm. The mean UPF value and UVA and UVB transmittance of GONF and RGONF coated fabrics were calculated according to the EN 13758-1:2001 [58, 59]. UPF was calculated as follows:


(2) $$\text{UPF}=\frac{\int_{290}^{400}{\text{E}}_{\lambda }\times {ɛ}_{\lambda }\times \text{d}\lambda }{\int_{290}^{400}{\text{E}}_{\lambda }\times {ɛ}_{\lambda }\times {\text{T}}_{\lambda }\times \text{d}\lambda }$$

where E_l_ is the solar irradiance, e_l_ is the erythema action spectrum, T_l_ is the spectral transmittance of the specimen (incoming light that passes through the specimen), dl is the wavelength increment (nm), and l is the wavelength (nm).

The UPF rating was calculated as follows:


(3) $$\text{UPF}={\text{UPF}}_{\text{average}}-{\text{t}}_{\alpha /2,\text{n}-1}\frac{\text{s}}{\sqrt{\text{n}}},$$

where s is the standard deviation, n is the number of the specimens and t_a/2,n-1_ is the table value for a = 0.05. A fabric having UPF rating higher than 40 (UPF 40+) can be labelled as UV protective. The durability of electrical surface resistivity, water contact angle and UV protection against to the washing was performed according to ISO 105 C06-A1S.

## 3. Results

### 3.1. Surface morphology and elemental analysis

**Figure 2:**
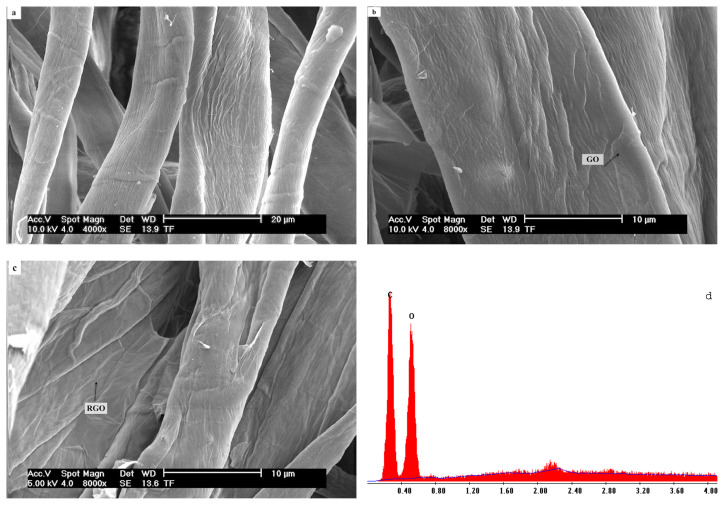
SEM images of cotton nonwoven fabric (**a)**, GONF70 (**b)**, RGONF70 (**c)**, EDS spectra of cotton nonwoven fabric (**d)** and RGONF70 (**e)**.

The SEM images of neat cotton nonwoven, GO coated cotton nonwoven and RGO coated cotton nonwoven fabric were presented in Figure 2. From Figure 2a, it can be seen that the surface of the neat cotton fibers are smooth and the typical longitudinal fibril structure with some convolutions along its length is evident. After coating with GO, the cotton fibers are covered by the GO sheets which appears as a wrinkled and rough surface on the fibers (Figure 2b). Some RGO sheets could be seen on cotton fibers after reduction process (Figure 2c). RGO sheets covered the cotton fibers and stacked to each other. The wrinkled and rough structure of RGO sheets on cotton fibers were remained, indicating the presence of reduced nature of GO sheets on fibers [60]. Moreover, the roughened surface composed of micropores is important for the hydrophobicity [61]. Furthermore, the elemental analysis of the neat cotton nonwoven fabric and RGO coated cotton nonwoven fabric were shown in Figures 2d and 2e, respectively. Also, the atomic and weight percentage ratios of neat cotton and RGO coated cotton nonwoven fabrics were presented in Table 2. The results show that the neat cotton nonwoven fabric composed of C and O having the atomic percentage ratio of 62.01% and 37.99%, respectively. However, the atomic percentage ratio of C and O for the cotton nonwoven fabric changed after the reduction process of GO. The atomic percentage ratio of C and O for the RGO coated nonwoven fabric was found as 68.44% and 31.56%, respectively. This results indicates that the C/O ratio increased with the reduction of GO, showing the formation of RGO sheets on cotton fibers.

### 3.2. FTIR spectroscopy

**Figure 3:**
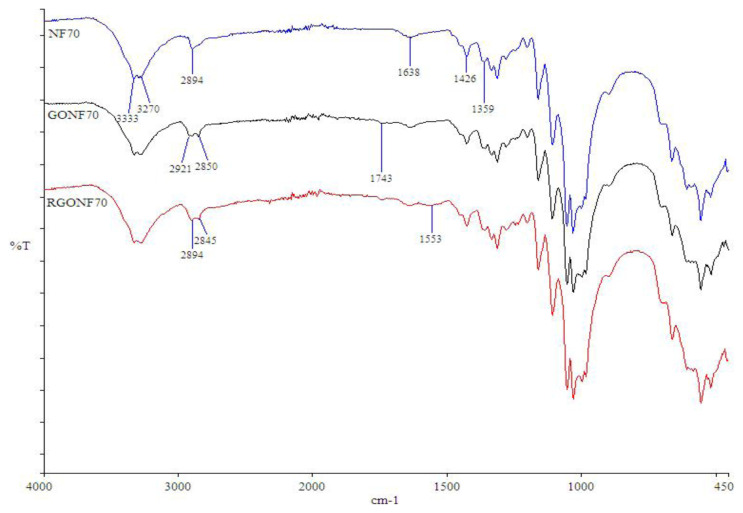
FTIR spectra of NF70, GONF70 and RGONF70.

The FTIR spectra of the neat cotton (NF70), GO coated (GONF70) and RGO coated (RGONF70) cotton nonwoven fabrics were given in Figure 3. The FTIR spectra of the neat cotton nonwoven fabric shows all the characteristic peaks of pure cotton, including hydrogen bonded OH stretching at 3333–3270 cm^−1^, asymmetrical C-H stretching at 2894 cm^−1^, O-H bending at 1638 cm^−1^, CH_2_ symmetric bending at 1426 cm^−1^ and the C-H bending at 1359 cm^−1^ [16, 62]. However, some changes in the FTIR spectra occurred after the GO coating. A new peak appears at the 1743 cm^−1^ which corresponds to C=O stretching arising from the carboxyl groups and the carbonyl containing groups in the GO [14, 62, 63]. The C-H stretching peak at 2894 cm^−1^ shifted to 2921 cm^−1^ and a new peak appeared at 2850 cm^−1^ because of the p-p conjugation between cotton and GO which leads to decreased bond strength. This indicates that the cellulose macromolecules chains were subjected to minor changes in intra and/or inter hydrogen bonding during GO coating and reduction processes [64, 65]. The peaks changed after the reduction process. The intensity of peak at 1638 cm^−1^ was decreased in the RGO coated cotton nonwoven fabric as compared to the neat cotton nonwoven fabric. Also, the peak at 1743 cm^−1^ became narrower than that of the GO coated cotton nonwoven fabric indicating the GO was successfully reduced into the RGO after the chemical reduction process with Na_2_S_2_O_4_ [16]. A broad peak appeared at 1553 cm^−1^ is attributed to C=C skeletal vibration of graphene [43].

### 3.3. Raman analysis

**Figure 4:**
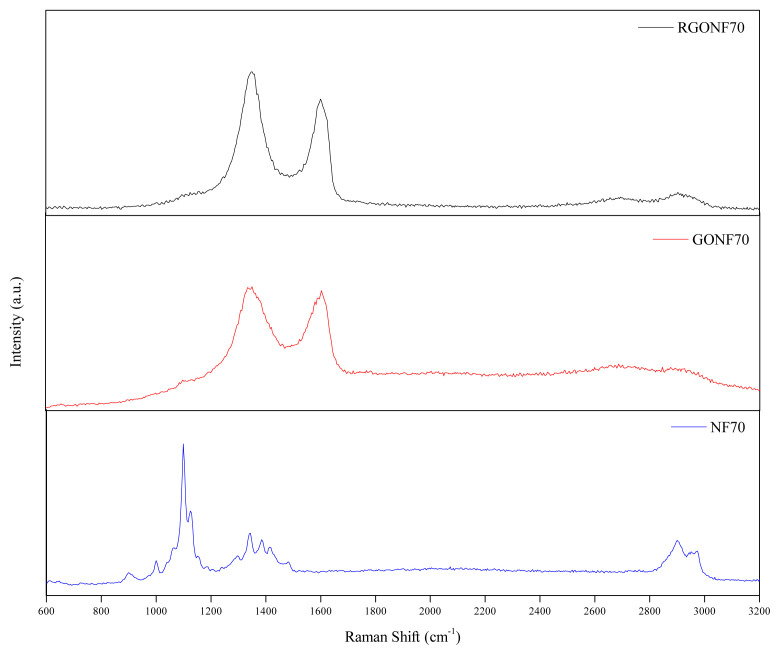
Raman spectra of NF70, GONF70 and RGONF70.

The structural changes before and after GO coating and after reduction process of GO were further characterized by Raman analysis. Raman spectra of cotton spunlace nonwoven fabric, GO and RGO coated sample are given in Figure 4. The spectrum of cotton spunlace nonwoven fabric (NF70) shows the characteristic peaks assigned to cotton. The strong peak at 1099 cm^−1^ is assigned to the asymmetric vibration of glycoside links in the cotton nonwoven fabric [66]. After GO coating process, the spectrum of cotton nonwoven changed prominently and the presence of GO nanosheets onto the cotton fibers can be easily differentiated by two characteristic peaks of carbon materials. The GONF70 and RGONF70 samples exhibit two prominent peaks correspond to the D band and G band, indicating the main characteristics of carbon materials, which reveal the coating of GO and RGO onto nonwoven fabric. From the spectra, it can be seen that the D band is located at 1351 cm^−1^ for both GONF70 and RGONF70 samples. G band is located at 1604 cm^−1^ for GONF70 and at 1600 cm^−1^ for RGONF70. The intensity ratio of D and G band (I_D_/I_G_) for GONF70 and RGONF70 was obtained as 1.02 and 1.07, respectively. The intensity of D band is higher than the G band of RGONF70 sample which reveals the presence of defects. It can be understand that not only the oxidation but also the chemical reduction process using sodium dithionite contributed to an increase of the structural disorder. The increase in I_D_/I_G_ ratio after reduction suggests a decrease in the average size of the sp^2^ domains upon the removal of oxygen containing functional groups [14, 67, 68].

### 3.4. Mechanical property

**Figure 5:**
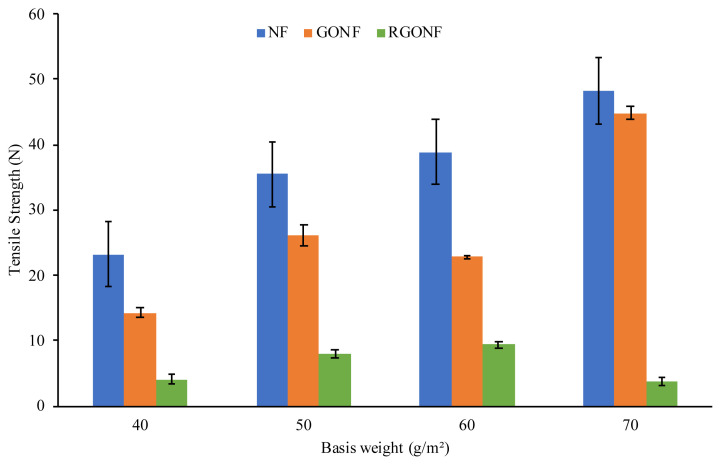
Tensile strength of cotton nonwoven, GO and RGO coated cotton nonwoven fabrics.

**Figure 6:**
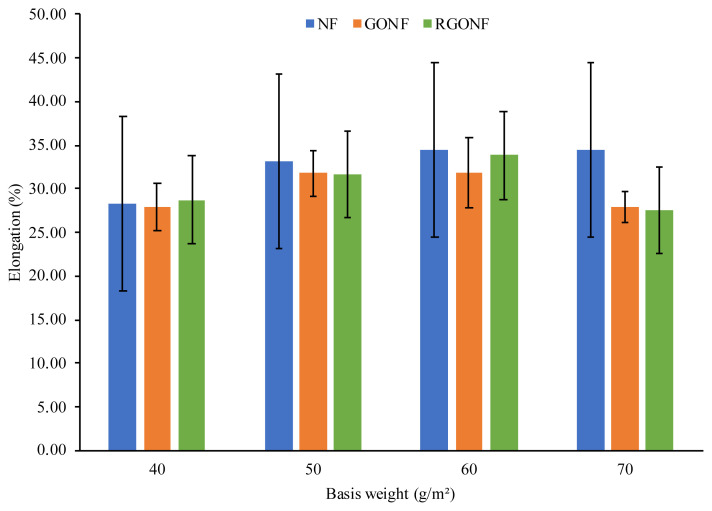
Elongation of cotton nonwoven, GO and RGO coated cotton nonwoven fabrics.

The mechanical performance of GO coated and RGO coated cotton nonwoven fabrics were tested in terms of tensile strength and elongation properties in the machine direction (MD). The detailed results are listed in Table 3. The tensile strength and elongation of the neat cotton nonwoven, GO coated cotton nonwoven and RGO coated cotton nonwoven fabrics is given in Figures 5 and 6, respectively. In Figure 5, it can be seen that the tensile strength of GO coated cotton nonwoven fabric decreased in comparison with the neat cotton nonwoven fabric. After the reduction process is applied to GO coated cotton nonwoven fabric, the decrease in tensile strength still continued. The highest tensile strength value was obtained at the GONF70 as 44.89 N showing percent change as low as 7.02%. The highest tensile strength value after reduction was obtained at the RGONF60 as 9.43 N. It can be seen from the tensile strength values of reduced samples, the mechanical performance of spunlace cotton nonwoven fabric coated with graphene oxide nanosheets was become weaker with the further reduction treatments. The explanation for the obtained results can be attributed to the poor bonding characteristics of the spunlace nonwoven structure. The basic building material that constitute the nonwoven fabric used in this study is the short staple cotton fibers and these fibers have become nonwoven fabric by mechanically bonding with the help of water jets. Since the structural integrity of the fabric is disrupted after each wet treatment, heating and mechanical effect applied, there has been a significant decrease in the mechanical properties.

On the other hand, the elongation of cotton spunlace nonwoven fabrics after GO coating showed little decrease at GONF40, GONF50 and GONF60 with percent change of 1.51%, 3.80% and 7.57%. The highest decrease in elongation after GO coating process is obtained at GONF70 with percent change of 19.28%. Besides, with the reduction of GO nanosheets coated on the cotton spunlace nonwoven fabrics, better elongation results were obtained. The elongation of RGONF40 and RGONF60 increased from 27.89% and 31.84% to 28.75% and 33.85%, respectively.

In order to determine the effect of GO coating and reduction process on the mechanical properties of cotton spunlace nonwoven fabric, statistical analysis was performed. One way ANOVA test was conducted for the mechanical properties of the nonwoven fabric samples at significance level of 0.05. Table 4 indicates the variance analysis results of GONF and RGONF samples. According to the results, GO coating and reduction process had significant effect on tensile strength of cotton spunlace nonwoven fabric. However, it was found out that the effect of GO coating of NF70 sample on the tensile strength was not statistically important. On the other hand, GO coating and reduction process were found out as no significant effect on elongation of cotton spunlace nonwoven fabric.

### 3.5. Color coordinates and reflectance measurements

**Figure 7:**
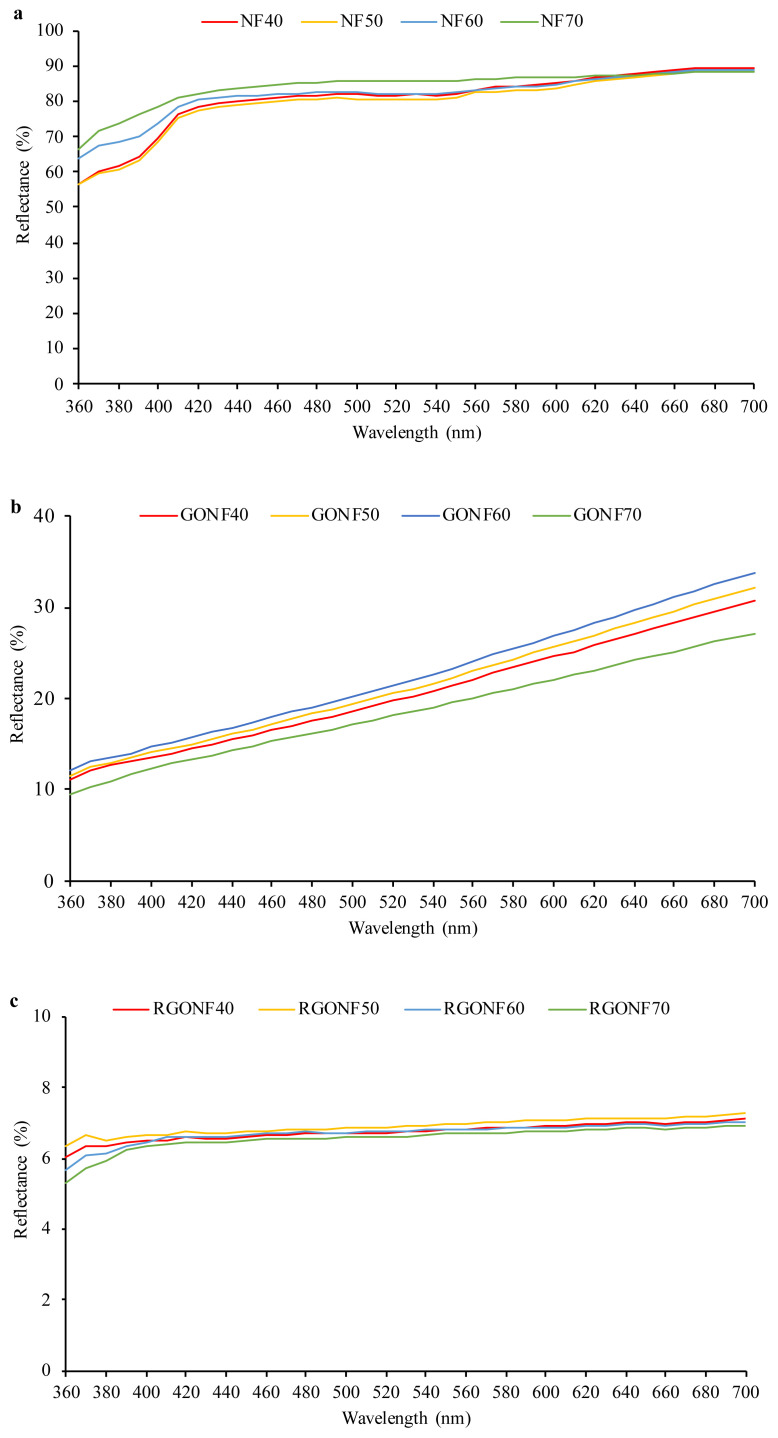
Reflectance spectra of the cotton nonwoven fabric **(a)**, GO coated cotton nonwoven fabric **(b)** and RGO coated cotton nonwoven fabric **(c)**.

As an easy way for determination of deposition and reduction of GO, the color of the fabric can be considered. The color changes of the cotton nonwoven fabrics after GO coating and reduction processes were determined by reflectance spectra and color coordinates. The reflectance spectra of cotton nonwoven fabric, GO and RGO coated cotton nonwoven fabrics within 360–700 nm are given in Figure 7. The reflectance spectra of the neat cotton nonwoven fabrics show that the fabrics reflect the most of the light in the visible region. The common characteristics for the neat cotton nonwoven fabrics are the high reflectance values across all wavelength which imply the materials are white. For the cotton nonwoven fabrics coated with GO, the reflectance curves of the fabrics shift to lower values which indicates that the most of the light is absorbed by the fabric and the color becomes darker. However, the spectra of GO coated nonwoven fabrics shows a tendency to increase with high wavelengths between 550 and 700 nm. This indicates that the GO coated cotton nonwoven fabrics are reflecting the light in yellow region (560–590 nm), orange region (590–620 nm) and red region (620–700 nm). In the case of RGO coated cotton nonwoven fabrics, the reflectance spectra show curves which are essentially flat at approximately 5% denoting the absorption of the light in all wavelengths. This reveals that RGO coated cotton spunlace fabrics absorb the most of the incident light without showing a distinct peak at the visible region. It can be understand that RGO coated cotton spunlace fabrics had dark color close to black.

The color coordinates and the color differences of the cotton nonwoven fabrics were given in Table 5. From the results, it can be seen that the cotton nonwoven fabrics have the highest L* values showing the bright whiteness, which increases with the increase in basis weight. After the coating process of cotton nonwoven fabrics with GO, the L* values decrease which indicates that the color of the nonwoven fabric becomes darker. The coating with GO changes the color of the nonwoven fabric from white to yellow-brown and this indicates the deposition of GO sheets on the cotton nonwoven fabric. According to the data, the L* value of the cotton nonwoven fabric with 70 g/m^2^ basis weight decreased from 94.35 to 51.41 after GO coating. There were also changes in a*, b* and C* values of fabrics. The coating of the cotton nonwoven fabrics with GO causes an increase in a* values towards redness with the increase in basis weight. Also, the significant increase in b* value indicates the increase in the yellowness. Furthermore, the b* value increases with the increase in basis weight from 40 to 70 g/m^2^ and the highest yellowness value is obtained with the highest basis weight which is GONF70. Also, the C* value increases for all GO coated nonwoven fabrics indicating that the color turned to yellow-brown. However, when the reduction process takes place, the a*, b* and C* values of all of the fabrics decrease dramatically. The L* value of the RGONF70 sample decreased to 31.07 indicating the blackness in the color. The a*, b* and C* values of the RGO coated cotton nonwoven fabrics decrease to almost zero which indicates that there are no dominant color and the fabrics are neutral grey. Additionally, the ΔE values show the color differences of the GO coated and reduced GO coated cotton nonwoven fabrics compared to neat cotton nonwoven fabric. The increased ΔE value of the GO coated nonwoven fabrics shows high degree of color difference from the neat cotton nonwoven fabric. However, after the reduction process the ΔE value of the RGONF70 sample increases to 63.284 which denotes the increase in the color difference compared to the neat cotton nonwoven fabric. In conclusion, the obtained data from the reflectance spectra and the color coordinates simultaneously show that the GO sheets are successfully and effectively deposited on the cotton nonwoven fabric and reduced.

### 3.6. Electrical surface resistance

**Figure 8:**
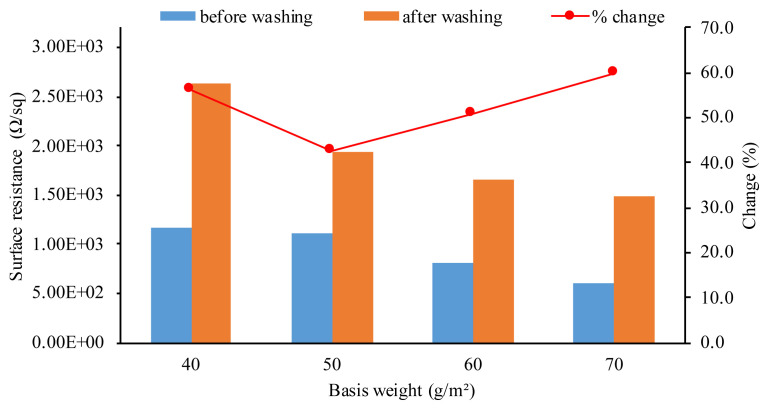
Electrical resistivity of RGO coated cotton nonwoven fabrics before and after washing.

The electrical surface resistance values of RGO coated cotton nonwoven fabrics before and after washing were given in Figure 8. The electrical conductivity is the prominent feature of the extent to which GO is transformed to RGO by the reduction process. The neat cotton and GO coated nonwoven fabrics have an insulating characteristic. After the reduction of GO coated nonwoven fabric by sodium dithionite, the surface resistance of fabrics as a function of basis weight was measured. The electrical surface resistance of RGO coated cotton nonwoven fabric before washing decreased from 1.16 × 10^3^ Ω/sq to 5.98 × 10^2^ Ω/sq by the increase of basis weight of the nonwoven fabrics from 40 g/m^2^ to 70 g/m^2^. The basis weight of the nonwoven fabric plays an important role for the enhancement of the electrical conductivity of RGO coated cotton nonwoven fabrics. The decrease in surface resistance could be explained by the increase of the nonwoven fabric weight. Nonwovens have porous structure which affects their mechanical, thermal and comfort properties. With the increase in basis weight and thickness of the nonwoven fabric, the more number of fibers participate in entanglement, thus, the interfiber space is closed. Also, basis weight affects the porosity of nonwoven fabric. The increase in basis weight causes the decrease in pore size of the spunlace nonwoven fabric [26]. Hence, the RGO sheets covers the surface of constituent fibers and inter-fiber spaces uniformly. Consequently, the decrease in surface resistance could be attributed to the effective recovery of the sp^2^ network of carbon via chemical reduction and good RGO sheet-to-sheet connection throughout the nonwoven fabric. In other words, RGO nanosheets form a continuous conductive thin layer on the surface of the spunlace nonwoven fabric to shorten the electron transfer pathways [64, 69].

**Figure 9:**
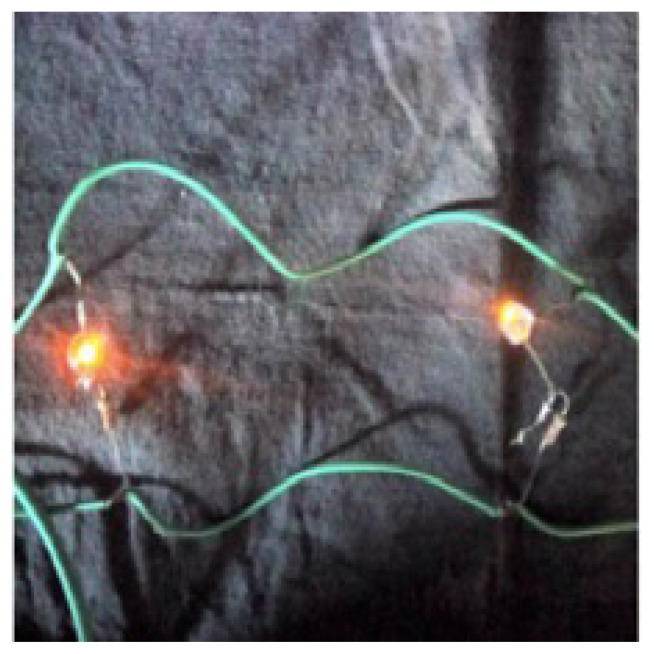
Digital photograph of a red LED light integrated with RGO coated cotton nonwoven fabric.

The electrical surface resistance of RGO coated nonwoven fabrics after washing increased slightly. The surface resistance of nonwoven fabrics after washing shows a similar trend like before washing. The increase in surface resistance after washing can be explained by the removal of loosely adsorbed RGO sheets. The change in percentage in surface resistance values of RGO coated cotton nonwoven fabrics before and after washing was also calculated. According to the results, the lowest % change was obtained with the nonwoven fabric having basis weight of 50 g/m^2^. This can be explained by the better coating of the GO sheets on the surface of cotton fibers, thus, less amount of RGO sheets removed during the washing process. However, the disruption of structural integrity of cotton spunlace fabric with wet processes has also affected the percentage change in surface electrical resistance. Due to the low dimensional stability and further loosening of the entanglement of staple cotton fibers after washing process, the interfiber space has widened, thus, the electrical pathway of RGO sheets was damaged. The reason of the higher % change in RGONF70 sample than the others could be the more deformation of dimensional stability and disruptions of fibers during wet processes. Besides, the lowest electrical surface resistance after washing was obtained as 1.49 × 10^3^ Ω/sq for RGONF70 sample. Furthermore, Figure 9 shows the electrical conductivity of RGO coated cotton nonwoven fabric by integrating with the red LED light. The RGO coated cotton nonwoven fabric has potential as an electrical conductor in various functional application areas.

### 3.7. Water contact angle

**Figure 10:**
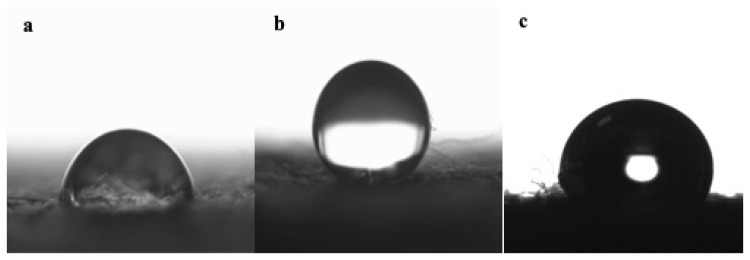
Water droplet images of GONF70 (**a)**, RGONF70 before (**b)** and after washing **(c)**.

**Figure 11:**
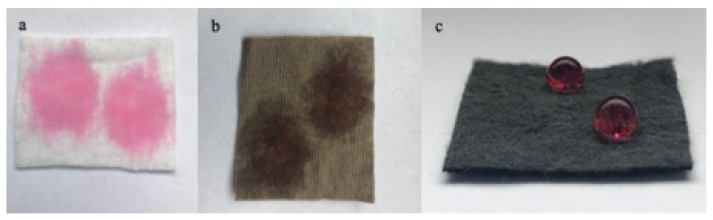
Digital image of colored water droplets on the cotton nonwoven fabric (NF70) (**a)**, GO coated nonwoven fabric (GONF70) (**b)**, RGO coated cotton nonwoven fabric (RGONF70) (**c)**.

Figure 10 shows the water contact angle of GO and RGO coated cotton nonwoven fabrics with basis weight of 70 g/m^2^. Figure 11 shows the digital photographs of the water droplets placed on the surface of the cotton nonwoven fabric, GO and RGO coated cotton nonwoven fabrics with basis weight of 70 g/m^2^. Cotton is hydrophilic in nature and has good water absorption property [16]. The neat cotton nonwoven fabric can be completely wetted by water due to the abundant hydroxyl groups in its structure (Figure 11) [7]. Also, the GO coated cotton nonwoven fabric shows a similar behavior due to the presence of sufficient oxygenated functional groups on the basal planes and edges of the GO sheets [70]. The GO coated cotton nonwoven fabric (Figure 10) shows hydrophilicity with an average water contact angle of 56.1°. When the reduction process performed, the change in water contact angle is remarkable. The water contact angle of RGO coated cotton nonwoven fabric increases to 108.2° with resulting hydrophobicity. The increase in water contact angle can be explained by the elimination of oxygen-containing functional groups after reduction. The phenomenon of the hydrophobicity can be referred as the contact angle value bigger than 90° [71]. After washing process, the water contact angle of RGONF70-W sample obtained as 106.6°. It is found out that the hydrophobic behavior of RGONF70 sample sustained after washing.

The water droplets placed on the surface of the RGO coated cotton nonwoven fabric are stable and could maintain their spherical shapes for a long period of time (Figure 11). Hydrophobic behavior of the RGO coated cotton nonwoven fabric can be explained by the removal of oxygen containing functional groups during reduction process.

### 3.8. UV protection property

**Figure 12:**
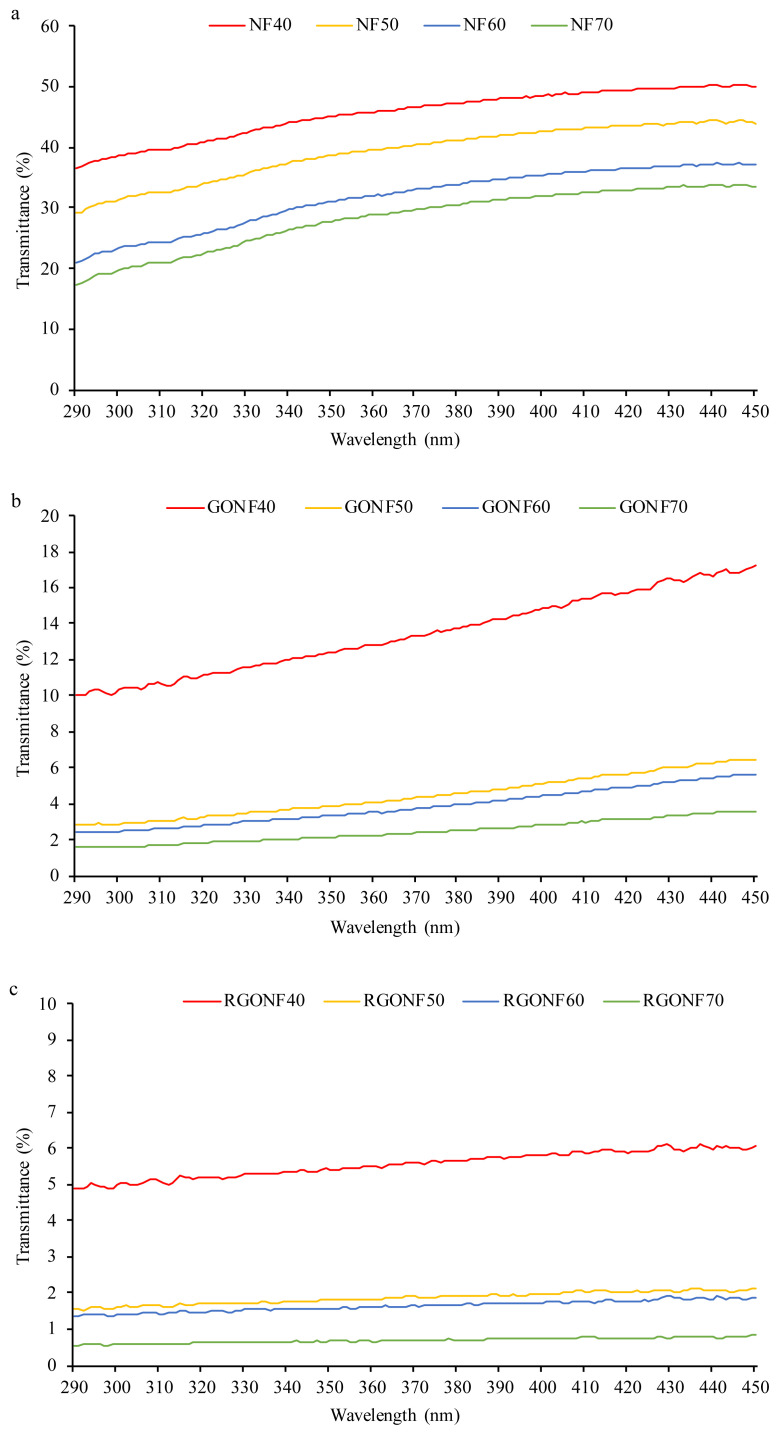
UV transmittance spectra of cotton nonwoven fabrics **(a)**, GO coated cotton nonwoven fabrics **(b)** and RGO coated nonwoven fabrics **(c)**.

The ultraviolet transmittance spectra of the cotton nonwoven fabrics, GO and RGO coated cotton nonwoven fabrics between 250 and 450 nm wavelength are given in Figure 12. In Figure 12a, the UV transmittance spectra of cotton spunlace nonwoven fabrics can be seen. The UV transmittance of neat cotton nonwoven fabrics has high values and gives different results depending on the basis weight. The highest UV transmittance was found at NF40 which has the lowest basis weight. With the increase in basis weight, UV transmittance decreases. Nevertheless, the UV transmittance of neat cotton spunlace nonwoven fabrics has high values throughout the UV region. Also, the UV transmittance curves of all fabrics show increasing trend with the increase of wavelength. The decline in UV transmittance can be seen in GO coated nonwoven fabric (Figure 12b). From the spectra, it is demonstrated that the coating of GO sheets acted as an effective material to block UV rays across fabric. UV rays can easily penetrate to the neat cotton nonwoven fabric whereas the penetration of UV rays are blocked considerably by the GO sheets on the cotton nonwoven fabric. Especially, GO coated cotton spunlace nonwoven fabrics with higher basis weights have lower UV transmittance percentage values in comparison with GO coated cotton spunlace nonwoven fabric with basis weight of 40 g/m^2^. The lowest UV transmittance values were obtained with GONF70. The UV transmittance spectra of RGO coated cotton spunlace nonwoven fabrics were given in Figure 12c. It can be seen that the homogenous coating of GO and successfully transformation of GO to reduced GO have affected the UV transmittance of the cotton spunlace nonwoven fabric in a positive way. Moreover, after the reduction process, the RGO coated cotton nonwoven fabric had much lower UV transmittance compared to neat and GO coated cotton nonwoven fabric. The attenuation in RGO coated cotton nonwoven fabric can be attributed to the UV absorption ability of reduced graphene oxide nanosheets. However, the basis weight of cotton spunlace nonwoven fabric has a prominent effect on UV transmittance. It can be obviously said that the higher the basis weight, the lower the UV transmittance. It is noteworthy to mention that the UV transmittance curves of the RGO coated cotton spunlace nonwoven fabrics became straighter without showing an increasing trend throughout the scanned UV region wavelengths.

**Figure 13:**
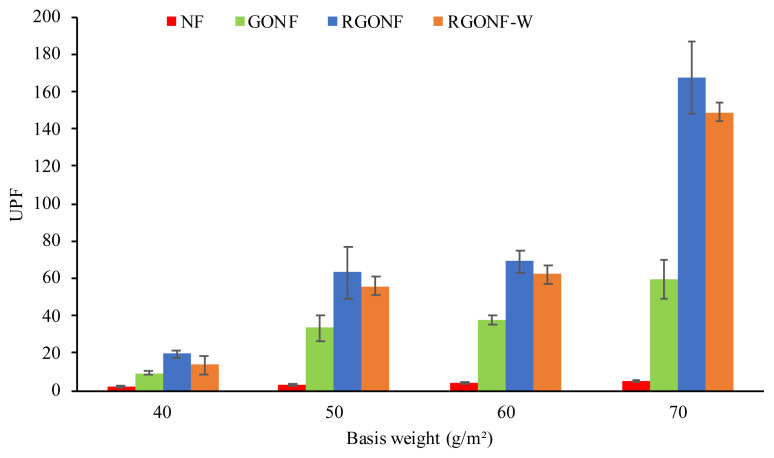
UPF values of cotton nonwoven fabrics, GO coated cotton nonwoven fabrics and RGO coated cotton nonwoven fabrics before and after washing as a function of basis weight.

In order to assess the degree of UV protection of cotton nonwoven, GONF, RGONF and RGONF-W samples as a function of the basis weight, the ultraviolet protection factor (UPF) was measured and given in Figure 13. The neat cotton spunlace nonwoven fabrics had the lowest UPF values. However, the UPF value showed a slight increase with the increase in basis weight of the cotton spunlace nonwoven fabrics. For the cotton nonwoven fabrics coated with GO, with the sequential increase in basis weight as 40, 50, 60 and 70 g/m^2^, the UPF values increased to 9.3, 33.35, 37.59 and 59.2, respectively. Fabric construction and composition are important parameters to the assessment of UV protection. As higher the basis weight of the nonwoven fabric, higher the thickness of the nonwoven fabric. The increase in thickness affects the ability of UV protection in a positive way. When the reduction process is applied to the GO coated cotton nonwoven fabrics, the highest UPF values as a function of basis weight were obtained. The UPF value of the RGONF40 is 19.77 while the UPF value of the RGONF70 is 167.34 which has an excellent protection with a UPF rating 40+ (according to the EN 13758-1:2001). After washing process, UPF values of RGONF samples showed a slight decrease. Also, UPF values of RGONF-W samples change in direct proportion with the increase in basis weight. The UPF values for RGONF40-W, RGONF50-W, RGONF60-W and RGONF70-W samples obtained as 13.81, 55.65, 62.15 and 149.18, respectively. The UPF ratings of cotton nonwoven, GONF, RGONF and RGONF-W samples as a function of basis weight according to the EN 13758-1:2001 standard are listed in Table 6. As it can be seen that RGO coated cotton spunlace nonwoven fabrics at higher basis weight which were coded as RGONF60 and RGONF70 have excellent UV protection property. After washing, the UPF ratings show slight decrease for RGONF40 and RGONF50 samples. On the other hand, the higher basis weight samples continued to show excellent UV protection after washing.

**Figure 14:**
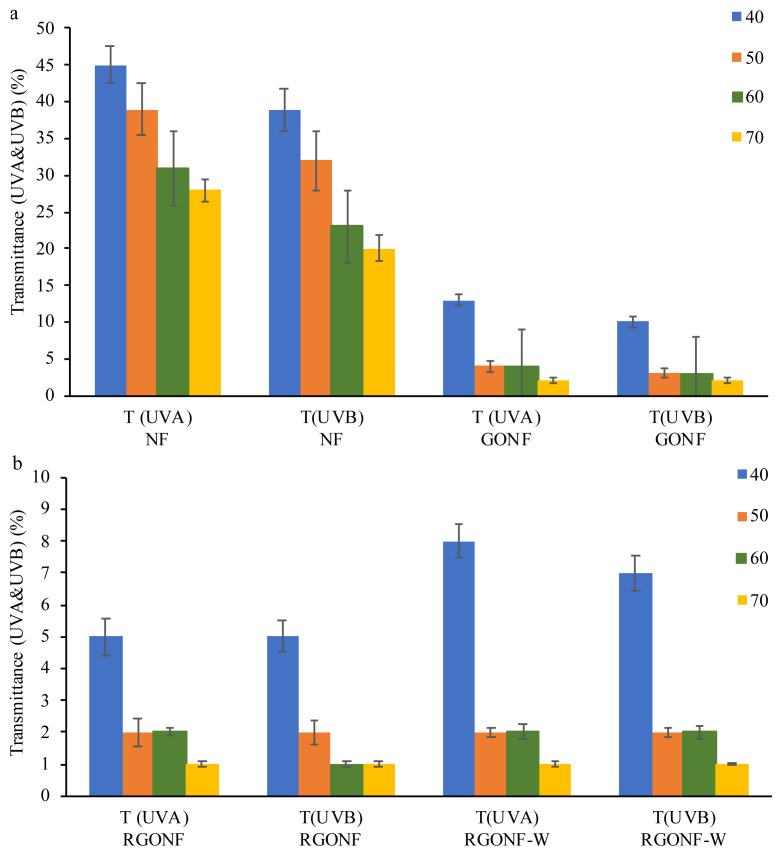
UVA & UVB transmittance of cotton nonwoven fabrics, GO coated cotton nonwoven fabrics (a) and RGO coated cotton nonwoven fabrics before and after washing (b) as a function of basis weight.

The transmittance of UVA and UVB rays of the cotton nonwoven, GONF, RGONF and RGONF-W samples were shown in Figure 14. The neat cotton nonwoven fabrics have the highest transmittance percentage for both UVA and UVB rays that decrease with the increase in basis weight. For the GO coated cotton nonwoven fabrics, the transmittance percentage for both UVA and UVB decreased prominently compared to the neat cotton nonwoven fabric (Figure 14a). Besides, the sharp decrease in transmittance percentage can be seen in samples GONF40 and GONF50 as a result of increase in basis weight. The decrease in transmittance percentages for both UVA and UVB rays further continued with increase of basis weight of cotton spunlace nonwoven fabric to 70 g/m^2^. On the other side, from Figure 14b it can be seen that the RGO coated cotton spunlace nonwoven fabrics had the lowest transmittance percentage in both UVA and UVB which imply the high level of blocking the penetration of UV rays through the fabrics. The UVA and UVB transmittance percentages of RGO coated cotton spunlace nonwoven fabrics were obtained below 5% for RGONF50, RGONF60 and RGONF70 and at 5% for RGONF40. After washing, the transmittance percentage of RGONF-W samples increased slightly. The lowest transmittance percentage was obtained with the highest basis weight sample in consistent with above mentioned results.

## 4. Discussion

Cotton spunlace nonwoven fabrics were successfully coated with GO nanosheets and chemical reduction process were applied with aqueous solution of sodium dithionite. The effect of basis weight of the cotton spunlace nonwoven fabrics on the properties of final material was investigated. Mechanical properties, color coordinates and color differences, surface electrical resistance, water contact angle and UV blocking property are studied. It is revealed that the basis weight of the cotton nonwoven fabrics has an improving effect on the surface electrical resistance and UV protection properties. The surface electrical resistance decreased by about 1.94 times with the increase in basis weight from 40 to 70 g/m^2^. The lowest surface electrical resistance was obtained at the RGO coated cotton nonwoven fabric with the highest basis weight (70 g/m^2^). After the washing process, the surface electrical resistance of all RGO coated cotton nonwoven fabrics increased and the lowest value is obtained at the 70 g/m^2^ basis weight nonwoven fabric with the percentage change in electrical resistance of 59.9%. Hydrophobicity was obtained with the reduction of GO. The UV transmittance of cotton spunlace nonwoven fabrics decreased prominently after the reduction of GO. Besides, UPF of cotton spunlace nonwoven fabrics increased with the reduction of GO and also, it is revealed that the UPF of RGO coated cotton nonwoven fabrics mainly affected by the basis weight of the initial cotton spunlace nonwoven fabric. The RGO coated cotton spunlace nonwoven fabric is a promising candidate with advantages such as low cost and easy processability for application areas such as wearable devices and smart textiles where flexibility, lightweight and functionality required.

## Figures and Tables

**Table 1:** Specifications of cotton nonwoven fabrics with corresponding sample codes.

Sample code	Basis weight (g/m^2^)	Tensile strength (MD) (N)	Elongation (%)
NF40	40	**23.21** ± 5	**28.32** ± 10
NF50	50	**35.54** ± 5	**33.10** ± 10
NF60	60	**38.94** ± 5	**34.45** ± 10
NF70	70	**48.28** ± 5	**34.54** ± 10

**Table 2:** Elemental analysis of cotton nonwoven and RGO coated nonwoven fabric obtained by EDS.

Sample	Atomic ratio (%)		Weight ratio (%)	
	Elements		Elements	
	C	O	C	O
**NF**	62.01	37.99	55.06	44.94
**RGONF**	68.44	31.56	61.95	38.05

**Table 3:** Tensile strength and elongation of GO and RGO coated cotton nonwoven fabrics.

Sample code	Tensile strength (MD) (N)	Tensile strength (MD) (cN/tex)	Elongation (%)
After coating			
GONF40	**14.30** ± 0.760	**0.64** ± 0.024	**27.89** ± 2.667
GONF50	**26.11** ± 1.543	**0.69** ± 0.010	**31.84** ± 2.613
GONF60	**22.86** ± 0.201	**0.65** ± 0.026	**31.84** ± 3.953
GONF70	**44.89** ± 1.077	**1.03** ± 0.026	**27.88** ± 1.769
**After reduction**	**Tensile strength (MD) (N)**	**Tensile strength (MD) (cN/tex)**	**Elongation (%)**
RGONF40	**4.13** ± 0.725	**0.21** ± 0.027	**28.75** ± 4.428
RGONF50	**8.03** ± 0.696	**0.28** ± 0.023	**31.65** ± 6.582
RGONF60	**9.43** ± 0.532	**0.32** ± 0.004	**33.85** ± 3.767
RGONF70	**3.82** ± 0.574	**0.11** ± 0.020	**27.58** ± 2.303

**Table 4:** One way ANOVA results for tensile strength and elongation of cotton nonwoven fabrics.

Source		Tensile strength (N)	Elongation (%)
**GONF**	40	**0,04** *	0,95
	50	**0,03** *	0,84
	60	**0,00** *	0,69
	70	0,28	0,32
**RGONF**	40	**0,00** *	0,95
	50	**0,00** *	0,84
	60	**0,00** *	0,93
	70	**0,00** *	0,31

* statistically important according to α = 0.05.

**Table 5:** Color coordinates and color differences of cotton nonwoven, GO coated cotton nonwoven and RGO coated cotton nonwoven fabrics.

Sample	L*	a*	b*	C*	h	X	Y	Z	ΔE
**NF40**	93.07	1.48	2.03	2.51	53.97	79.56	83.13	86.36	-
**NF50**	92.57	1.50	2.00	2.50	53.10	78.48	81.99	85.20	-
**NF60**	93.17	1.33	1.33	1.88	45.18	79.71	83.37	87.58	-
**NF70**	94.35	0.11	1.44	1.45	85.53	81.67	86.08	90.29	-
**GONF40**	53.62	3.76	11.17	11.78	71.40	21.28	21.62	17.31	40.558
**GONF50**	54.57	3.88	11.39	12.04	71.20	22.17	22.51	17.99	39.220
**GONF60**	55.61	4.04	11.58	12.27	70.79	23.20	23.53	18.79	39.028
**GONF70**	51.41	3.17	10.26	10.74	72.83	19.22	19.62	15.96	43.940
**RGONF40**	31.36	0.29	0.76	0.81	68.90	6.48	6.80	7.10	61.742
**RGONF50**	31.71	0.28	0.79	0.84	70.56	6.62	6.96	7.25	60.886
**RGONF60**	31.36	0.19	0.56	0.59	71.34	6.47	6.81	7.16	61.827
**RGONF70**	31.07	0.21	0.72	0.75	73.48	6.35	6.68	6.98	63.284

**Table 6:** UPF ratings of cotton nonwoven fabrics, GO coated cotton nonwoven fabrics and RGO coated cotton nonwoven fabrics as a function of basis weight.

Sample code	UPF rating (EN 13758-1:2001)			
	NF	GONF	RGONF	RGONF-W
**40**	2.32	8.61	17.61	12.74
**50**	2.67	26.56	48.19	47.16
**60**	3.71	34.67	50+	50+
**70**	4.27	48.45	50+	50+

## References

[1] Gültekin BC Gültekin ND Atak O Şimşek R Evaluation of the Electromagnetic Shielding Effectiveness of Carbon-Based Screen Printed Polyester Fabrics Fibers and Polymers 2018 19 2 313 320 10.1007/s12221-018-7462-7

[2] Knittel D Schollmeyer E Electrically high-conductive textiles Synthetic Metals 2009 159 14 1433 1437 10.1016/j.synthmet.2009.03.021

[3] Xue C-H Chen J Yin W Jia S-T Ma J-Z Superhydrophobic conductive textiles with antibacterial property by coating fibers with silver nanoparticles Applied Surface Science 2012 258 7 2468 2472 10.1016/j.apsusc.2011.10.074

[4] Sharaf S Higazy A Hebeish A Propolis induced antibacterial activity and other technical properties of cotton textiles International Journal of Biological Macromolecules 2013 59 408 16 10.1016/j.ijbiomac.2013.04.030 23665479

[5] Gültekin BC Akalin M Yükseloğlu SM The study of flame retardancy and thermal properties of SeaCell® fabrics TEKSTİL ve KONFEKSİYON 2013 23 2 107 112 doi:

[6] Yang H Yang CQ Durable flame retardant finishing of the nylon/cotton blend fabric using a hydroxyl-functional organophosphorus oligomer Polymer Degradation and Stability 2005 88 3 363 370 10.1016/j.polymdegradstab.2004.11.013

[7] Shateri Khalil-Abad M Yazdanshenas ME Superhydrophobic antibacterial cotton textiles Journal of Colloid and Interface Science 2010 351 1 293 298 10.1016/j.jcis.2010.07.049 20709327

[8] Hoefnagels HF Wu D de With G Ming W Biomimetic Superhydrophobic and Highly Oleophobic Cotton Textiles Langmuir 2007 23 13158 13163 doi: 17985939 10.1021/la702174x

[9] Tang X Tian M Qu L Zhu S Guo X Functionalization of cotton fabric with graphene oxide nanosheet and polyaniline for conductive and UV blocking properties Synthetic Metals 2015 202 82 88 10.1016/j.synthmet.2015.01.017

[10] Xin JH Daoud WA Kong YY A New Approach to UV-Blocking Treatment for Cotton Fabrics Textile Research Journal 2004 74 2 97 100 10.1177/004051750407400202

[11] Ramadoss A Saravanakumar B Kim SJ Thermally reduced graphene oxide-coated fabrics for flexible supercapacitors and self-powered systems Nano Energy 2015 15 587 597 10.1016/j.nanoen.2015.05.009

[12] Hu XL Tian MW Qu LJ Zhu SF Han GT Multifunctional cotton fabrics with graphene/polyurethane coatings with far-infrared emission, electrical conductivity, and ultraviolet-blocking properties Carbon 2015 95 625 633 10.1016/j.carbon.2015.08.099

[13] Shen C Kao T Huang C Lee J Wearable Band Using a Fabric-Based Sensor for Exercise ECG Monitoring 2006 10th IEEE International Symposium on Wearable Computers 2006 143 144

[14] Sahito IA Sun KC Arbab AA Qadir MB Jeong SH Integrating high electrical conductivity and photocatalytic activity in cotton fabric by cationizing for enriched coating of negatively charged graphene oxide Carbohydrate Polymers 2015 130 299 306 10.1016/j.carbpol.2015.05.010 26076630

[15] Hasani M Montazer M Electro-conductivity, bioactivity and UV protection of graphene oxide-treated cellulosic/polyamide fabric using inorganic and organic reducing agents The Journal of The Textile Institute 2017 108 10 1777 1786 10.1080/00405000.2017.1286700

[16] Cai G Xu Z Yang M Tang B Wang X Functionalization of cotton fabrics through thermal reduction of graphene oxide Applied Surface Science 2017 393 441 448 10.1016/j.apsusc.2016.10.046

[17] Di Y Li Q Zhuang X Antibacterial Finishing of Tencel/Cotton Nonwoven Fabric Using Ag Nanoparticles-Chitosan Composite Journal of Engineered Fibers and Fabrics 2012 7 2 155892501200700205 10.1177/155892501200700205

[18] Asadi M Montazer M Multi-functional Polyester Hollow Fiber Nonwoven Fabric with Using Nano Clay/Nano TiO2/Polysiloxane Composites Journal of Inorganic and Organometallic Polymers and Materials 2013 23 6 1358 1367 10.1007/s10904-013-9937-3

[19] Pan Y-J Lou C-W Hsieh C-T Huang C-H Lin Z-I Nonwoven fabric/spacer fabric/polyurethane foam composites: Physical and mechanical evaluations Fibers and Polymers 2016 17 5 789 794 10.1007/s12221-016-5736-0

[20] Erdem R Rajendran S Influence of Silver Loaded Antibacterial Agent on Knitted and Nonwoven Fabrics and Some Fabric Properties Journal of Engineered Fibers and Fabrics 2016 11 1 155892501601100107 10.1177/155892501601100107

[21] Akalin M Yukseloglu SM Gultekin BC Agirgan AO Novel Approach to Breathable Nonwoven Hygienic Products Anand SC Kennedy JF Miraftab M Rajendran S Medical Textiles and Biomaterials for Healthcare Woodhead Publishing 2006 201 208

[22] Thangadurai K Thilagavathi G Bhattacharyya A Characterization of needle-punched nonwoven fabrics for industrial air filter application The Journal of The Textile Institute 2014 105 12 1319 1326 10.1080/00405000.2014.895089

[23] Ozen MS Sancak E Beyit A Usta I Akalin M Investigation of electromagnetic shielding properties of needle-punched nonwoven fabrics with stainless steel and polyester fiber Textile Research Journal 2012 10.1177/0040517512461683

[24] Ozen MS Sancak E Soin N Shah TH Siores E Investigation of electromagnetic shielding effectiveness of needle punched nonwoven fabric produced from conductive silver coated staple polyamide fibre The Journal of The Textile Institute 2015 107 7 912 922 10.1080/00405000.2015.1070604

[25] Ghali L Halimi MT Hassen MB Sakli F Effect of Blending Ratio of Fibers on the Properties of Nonwoven Fabrics Based of Alfa Fibers Advances in Materials Physics and Chemistry 2014 4 6 10 10.4236/ampc.2014.46014

[26] Jain RK Sinha SK Das A Structural investigation of spunlace nonwoven Research Journal of Textile and Apparel 2018 22 3 158 179 10.1108/RJTA-07-2017-0038

[27] Maiti S Bele VS Basu SK Effect of material properties and process parameters on properties of hydroentangled nonwoven fabrics The Journal of The Textile Institute 2021 112 6 914 920 10.1080/00405000.2020.1791398

[28] Gültekin E Çelik Hİ Nohut S Elma SK Predicting air permeability and porosity of nonwovens with image processing and artificial intelligence methods The Journal of The Textile Institute 2020 111 11 1641 1651 10.1080/00405000.2020.1727267

[29] Yang J Pu Y He H Cao R Miao D Superhydrophobic cotton nonwoven fabrics through atmospheric plasma treatment for applications in self-cleaning and oil–water separation Cellulose 2019 26 12 7507 7522 10.1007/s10570-019-02590-y

[30] Singh V Joung D Zhai L Das S Khondaker SI Graphene based materials: Past, present and future Progress in Materials Science 2011 56 8 1178 1271 10.1016/j.pmatsci.2011.03.003

[31] Zhang L Liang J Huang Y Ma Y Wang Y Size-controlled synthesis of graphene oxide sheets on a large scale using chemical exfoliation Carbon 2009 47 14 3365 3368 10.1016/j.carbon.2009.07.045

[32] Zhong Y Zhen Z Zhu H Graphene: Fundamental research and potential applications FlatChem 2017 4 20 32 10.1016/j.flatc.2017.06.008

[33] Stobinski L Lesiak B Malolepszy A Mazurkiewicz M Mierzwa B Graphene oxide and reduced graphene oxide studied by the XRD, TEM and electron spectroscopy methods Journal of Electron Spectroscopy and Related Phenomena 2014 195 145 154 10.1016/j.elspec.2014.07.003

[34] Ren S Rong P Yu Q Preparations, properties and applications of graphene in functional devices: A concise review Ceramics International 2018 44 11 11940 11955 10.1016/j.ceramint.2018.04.089

[35] Li F Jiang X Zhao J Zhang S Graphene oxide: A promising nanomaterial for energy and environmental applications Nano Energy 2015 16 488 515 10.1016/j.nanoen.2015.07.014

[36] Cheng H Hu C Zhao Y Qu L Graphene fiber: a new material platform for unique applications NPG Asia Materials 2014 6 7 e113 e113 10.1038/am.2014.48

[37] Tissera ND Wijesena RN Perera JR de Silva KMN Amaratunge GAJ Hydrophobic cotton textile surfaces using an amphiphilic graphene oxide (GO) coating Applied Surface Science 2015 324 455 463 10.1016/j.apsusc.2014.10.148

[38] Abdelkader AM Karim N Vallés C Afroj S Novoselov KS Ultraflexible and robust graphene supercapacitors printed on textiles for wearable electronics applications 2D Materials 2017 4 3 035016 10.1088/2053-1583/aa7d71

[39] Karim N Afroj S Malandraki A Butterworth S Beach C All inkjet-printed graphene-based conductive patterns for wearable e-textile applications Journal of Materials Chemistry C 2017 5 44 11640 11648 10.1039/c7tc03669h

[40] Fugetsu B Sano E Yu H Mori K Tanaka T Graphene oxide as dyestuffs for the creation of electrically conductive fabrics Carbon 2010 48 12 3340 3345 10.1016/j.carbon.2010.05.016

[41] Ouadil B Cherkaoui O Safi M Zahouily M Surface modification of knit polyester fabric for mechanical, electrical and UV protection properties by coating with graphene oxide, graphene and graphene/silver nanocomposites Applied Surface Science 2017 414 292 302 10.1016/j.apsusc.2017.04.068

[42] Mengal N Sahito IA Arbab AA Sun KC Qadir MB Fabrication of a flexible and conductive lyocell fabric decorated with graphene nanosheets as a stable electrode material Carbohydrate Polymers 2016 152 19 25 10.1016/j.carbpol.2016.06.099 27516245

[43] Xu LL Guo MX Liu S Bian SW Graphene/cotton composite fabrics as flexible electrode materials for electrochemical capacitors RSC Advances 2015 5 32 25244 25249 10.1039/c4ra16063k

[44] Tian M Hu X Qu L Du M Zhu S Ultraviolet protection cotton fabric achieved via layer-by-layer self-assembly of graphene oxide and chitosan Applied Surface Science 2016 377 141 148 10.1016/j.apsusc.2016.03.183

[45] Mizerska U Fortuniak W Makowski T Svyntkivska M Piorkowska E Electrically conductive and hydrophobic rGO-containing organosilicon coating of cotton fabric Progress in Organic Coatings 2019 137 10.1016/j.porgcoat.2019.105312

[46] Du D Li P Ouyang J Graphene coated nonwoven fabrics as wearable sensors Journal of Materials Chemistry C 2016 4 15 3224 3230 10.1039/c6tc00350h

[47] Zhou X Song W Zhu G A facile approach for fabricating silica dioxide/reduced graphene oxide coated cotton fabrics with multifunctional properties Cellulose 2020 27 5 2927 2938 10.1007/s10570-020-02990-5

[48] Božič M Kokol V Ecological alternatives to the reduction and oxidation processes in dyeing with vat and sulphur dyes Dyes and Pigments 2008 76 2 299 309 10.1016/j.dyepig.2006.05.041

[49] Roessler A Jin X State of the art technologies and new electrochemical methods for the reduction of vat dyes Dyes and Pigments 2003 59 3 223 235 10.1016/S0143-7208(03)00108-6

[50] Meksi N Ben Ticha M Kechida M Mhenni MF Using of ecofriendly α-hydroxycarbonyls as reducing agents to replace sodium dithionite in indigo dyeing processes Journal of Cleaner Production 2012 24 149 158 10.1016/j.jclepro.2011.11.062

[51] Shateri-Khalilabad M Yazdanshenas ME Fabricating electroconductive cotton textiles using graphene Carbohydrate Polymers 2013 96 1 190 5 10.1016/j.carbpol.2013.03.052 23688469

[52] Molina J Fernández J del Río AI Bonastre J Cases F Chemical and electrochemical study of fabrics coated with reduced graphene oxide Applied Surface Science 2013 279 46 54 10.1016/j.apsusc.2013.04.020

[53] Molina J Fernández J Inés JC del Río AI Bonastre J Electrochemical characterization of reduced graphene oxide-coated polyester fabrics Electrochimica Acta 2013 93 44 52 10.1016/j.electacta.2013.01.071

[54] Cao J Wang C Multifunctional surface modification of silk fabric via graphene oxide repeatedly coating and chemical reduction method Applied Surface Science 2017 405 380 388 10.1016/j.apsusc.2017.02.017

[55] Cao J Wang C Highly conductive and flexible silk fabric via electrostatic self assemble between reduced graphene oxide and polyaniline Organic Electronics 2018 55 26 34 10.1016/j.orgel.2017.12.016

[56] Gültekin N Usta İ Yalçin B Green Reduction of Graphene Oxide Coated Polyamide Fabric Using Carob Extract AATCC Journal of Research 2020 7 6 33 40 10.14504/ajr.7.6.5

[57] Ajmeri JR Ajmeri CJ Developments in nonwoven materials for medical applications Kellie G Advances in Technical Nonwovens Duxford, CB22 4QH, UK Woodhead Publishing 2016 227 256

[58] Gambichler T Laperre J Hoffmann K The European standard for sun-protective clothing: EN 13758 Journal of the European Academy of Dermatology and Venereology 2006 20 2 125 130 doi: 16441617 10.1111/j.1468-3083.2006.01401.x

[59] Laperre J Foubert F European Standards for Protective Apparel Against UV Radiation Dummer R Nestle FO Burg G Cancers of the Skin Proceedings of the 8th World Congress Heidelberg, Berlin, Germany Springer, Berlin 2002 35 41 10.1007/978-3-642-59410-6_5 12079233

[60] Pandiyarasan V Archana J Pavithra A Ashwin V Navaneethan M Hydrothermal growth of reduced graphene oxide on cotton fabric for enhanced ultraviolet protection applications Materials Letters 2017 188 123 126 10.1016/j.matlet.2016.11.047

[61] Dashairya L Rout M Saha P Reduced graphene oxide-coated cotton as an efficient absorbent in oil-water separation Advanced Composites and Hybrid Materials 2017 1 1 135 148 10.1007/s42114-017-0019-9

[62] Sahito IA Sun KC Arbab AA Qadir MB Jeong SH Graphene coated cotton fabric as textile structured counter electrode for DSSC Electrochimica Acta 2015 173 164 171 10.1016/j.electacta.2015.05.035

[63] Bhattacharjee S Macintyre CR Wen X Bahl P Kumar U Nanoparticles incorporated graphene-based durable cotton fabrics Carbon 2020 166 148 163 10.1016/j.carbon.2020.05.029

[64] Amesimeku J Song W Wang C Fabrication of electrically conductive and improved UV-resistant aramid fabric via bio-inspired polydopamine and graphene oxide coating The Journal of The Textile Institute 2019 110 10 1484 1492 10.1080/00405000.2019.1607453

[65] Liu Y Liu L Li Z Zhao Y Yao J Green and facile fabrication of smart cellulose composites assembled by graphene nanoplates for dual sensing Cellulose 2019 26 17 9255 9268 10.1007/s10570-019-02735-z

[66] Zhao J Deng B Lv M Li J Zhang Y Graphene oxide-based antibacterial cotton fabrics Advanced Healthcare Materials 2013 2 9 1259 66 10.1002/adhm.201200437 23483725

[67] Chen X Meng D Wang B Li B-W Li W Rapid thermal decomposition of confined graphene oxide films in air Carbon 2016 101 71 76 10.1016/j.carbon.2016.01.075

[68] Lavin-Lopez MP Paton-Carrero A Sanchez-Silva L Valverde JL Romero A Influence of the reduction strategy in the synthesis of reduced graphene oxide Advanced Powder Technology 2017 28 12 3195 3203 10.1016/j.apt.2017.09.032

[69] Ren J Wang C Zhang X Carey T Chen K Environmentally-friendly conductive cotton fabric as flexible strain sensor based on hot press reduced graphene oxide Carbon 2017 111 622 630 10.1016/j.carbon.2016.10.045

[70] Shateri-Khalilabad M Yazdanshenas ME Preparation of superhydrophobic electroconductive graphene-coated cotton cellulose Cellulose 2013 20 2 963 972 10.1007/s10570-013-9873-y

[71] Das S Bhowmick M Chattopadhyay SK Basak S Application of biomimicry in textiles Current Science 2015 109 5 893 901 10.18520/vl09/i5/893-901

